# Influence of Three Different Surgical Techniques on Microscopic Damage of Saphenous Vein Grafts—A Randomized Study

**DOI:** 10.3390/medicina59020217

**Published:** 2023-01-23

**Authors:** Igor Zivkovic, Stasa Krasic, Milica Stankovic, Petar Milacic, Aleksandar Milutinovic, Djordje Zdravkovic, Zoran Tabakovic, Miodrag Peric, Miljan Krstic, Milovan Bojic, Dragan Milic, Slobodan Micovic

**Affiliations:** 1 Cardiac Surgery Department, Dedinje Cardiovascular Institute, 11000 Belgrade, Serbia; 2 School of Medicine, University of Belgrade, 11000 Belgrade, Serbia; 3 Cardiology Department, Mother and Child Health Care Institute, 11000 Belgrade, Serbia; 4 Center for Pathology and Pathological Anatomy, Clinical Center of Niš, 18000 Niš, Serbia; 5 Faculty of Medicine, University of Niš, 18000 Niš, Serbia; 6 Cardiac Surgery Department, Clinical Center of Niš, 18000 Niš, Serbia

**Keywords:** CABG, conventional vein harvesting, endoscopic vein harvesting, no-touch vein harvesting, histology, immunohistochemistry

## Abstract

*Background and Objectives:* The saphenous vein is one of the most common used grafts (SVG) for surgical revascularization. The mechanism of the SVGs occlusion is still unknown. Surgical preparation techniques have an important role in the early and late graft occlusion. Our study analyzed the influence of the three different surgical techniques on the histological and immunohistochemical characteristics of the vein grafts. *Methods:* Between June 2019 and December 2020, 83 patients who underwent surgical revascularization were prospectively randomly assigned to one of the three groups, according to saphenous vein graft harvesting (conventional (CVH), no-touch (NT) and endoscopic (EVH)) technique. The vein graft samples were sent on the histological (hematoxylin-eosin staining) and immunohistochemical (CD31, Factor VIII, Caveolin and eNOS) examinations. *Results:* The CVH, NT, and EVH groups included 27 patients (mean age 67.66 ± 5.6), 31 patients (mean age 66.5 ± 7.4) and 25 patients (mean age 66 ± 5.5), respectively. Hematoxylin-eosin staining revealed a lower grade of microstructural vein damage in the NT group (2, IQR 1-2) in comparison with CVH and EVH (3, IQR 2-4), (4, IQR 2-4) respectively (*p* < 0.001). Immunohistochemical examination revealed a high grade of staining in the NT group compared to the CVH and EVH group (CD 31 antibody *p* = 0.02, FVIII, *p* < 0.001, Caveolin, *p* = 0.001, and eNOS, *p* = 0.003). *Conclusion:* The best preservation of the structural vein integrity was in the NT group, while the lowest rate of leg wound complication was in the EVH group. These facts increase the interest in developing and implementing the endoscopic no-touch technique.

## 1. Introduction

Coronary artery bypass grafting (CABG) is one of the most common treatment options for multiple coronary artery disease (CAD). This procedure significantly increases the survival rate and quality of the life of the patients with CAD [1]. Although arterial grafts have been used frequently in the last decade, saphenous vein grafts (SVG) are still the most used conduit. According to the Society of thoracic surgeon’s database, approximately 89.3% of CABG was performed using the internal thoracic artery and SVG, while the rest of the procedures were performed with a different combination of arterial grafts [2,3]. The main disadvantage of SVG is a considerably high rate of graft failure. The graft patency at the 5- and 10-years range is between 75% and 86 %, and 55% and 60%, respectively [4]. The mechanism of the SVGs occlusion is still unknown. Numerous factors are known as contributors to the SVGs failure, including graft, vessels diameter, surgical conduit preparation, conduit handling, grafting site, coronary target vessels (run-off), patient risk factors, surgical skills techniques, and technical errors [5]. Recent evidence showed that the harvesting method, surgeon skill and experience had a meaningful impact on the graft patency rate [6]. Blunt surgical trauma and excessive manipulation decrease the endothelial integrity and function, and induce vein graft failure due to thrombosis, intimal hyperplasia, and atherosclerosis in the early, intermediate, and late periods, respectively [7].

Different SVG harvesting techniques were developed to reduce postoperative complications and preserve the structural integrity of the SVGs [7].

We analyzed the influence of the three different harvesting techniques on the histological and immunohistochemical characteristics of the SVGs.

## 2. Methods

### 2.1. Study Design

Institutional Review board approval: No-655, 13. January 2019.

Ethics Committee, Faculty of medicine, University of Nis: No-12-3340-2/6, 26. May 2019.

The study was approved by the institutional review board and the ethics committee of the medical faculty. Informed consent was obtained from all participants. The patients who underwent isolated CABG were randomly assigned to one of the three groups depending on vein harvesting techniques: conventional (CVH), no-touch (NT) and endoscopic (EVH) approaches.

#### Participants and Randomization

The patients who underwent open-chest CABG, with at least one SVG, were candidates for study. The patients with the inclusion criteria were identified and assessed for eligibility. Concomitant cardiac procedures, urgent procedures, REDO procedures, significant peripheral vascular disease, and patients planned for multiple arterial revascularizations were excluded from the study. Methods I in the data supplement describes additional explanations of the inclusion and exclusion criteria.

Preoperative saphenous vein mapping on both legs was performed in all patients. Ultrasound examination analyzed vein diameter, varies existence, vein coursing and the position of perforators. The mapping protocol is completely presented in Methods II in the data supplement.

Patients eligible for the study, who gave informed consent on the procedure day, were randomized into three groups. The simple randomization was achieved using computer-generated random numbers.

### 2.2. Surgical Procedure

All grafts in the study were prepared by one resident of cardiac surgery, who harveste more than 100 vein grafts per year.

Vein grafts were harvested through the longitudinal skin and subcutaneous incision, using the conventional and no-touch techniques. According to preoperative ultrasound mapping, we used the best part of the saphenous vein. In the conventional technique, perivascular tissue and fascia were sharply dissected with scissors, and a syringe with a manometer distended (pressure no more than 200 mmHg) the grafts with heparinized saline solution. In the NT group, grafts were harvested with intact perivascular tissue, fascia, adventitia, and fat pedicle. Syringe distension was not performed in this group; distension and a check for bleeding were performed using a catheter connected to the aortic cannula. The endoscopic technique was performed through the 1.5cm long skin incision using the VirtuoSaph plus endoscopic vessel harvesting open CO_2_ system by Terumo cardiovascular system corporation (125 Blue Ball Road, Elkton, Maryland 21921-5315). According to protocol, the harvesting process was performed without systemic heparinization. After finishing the harvesting process, vein grafts were distended by a syringe with a manometer (pressure no more than 200 mmHg) using heparinized saline solution. In the endoscopic group, only the skin suture was used. Methods III in the Supplementary Materials shows the full details of the surgical procedures.

### 2.3. Pathohistological Protocol

The 1 cm long vein sample was taken from all patients immediately after finishing the harvesting and before vein distension by syringe (in CVH and EVH groups). The saphenous vein samples were fixed in a 4% buffered formalin solution (immersion fixation) and molded into paraffin. The paraffin blocks were sectioned at a thickness of 4 µm, then deparaffinized, rehydrated, and stained with hematoxylin and eosin (H&E), followed by standard protocols. Two independent pathologists observed all vein samples for the structure changes with a detailed examination of the entire H&E section for each patient. The extent of the vein wall changes was based on the estimation of all three layers: tunica intima, media, and adventitia (Supplement Figures S1–S3). All tissue slides were examined and scored according to the structural damage: Grade 0—absent, Grade 1—minimal, Grade 2—mild, Grade 3—moderate, Grade 4—marked, Grade 5—very marked, Grade 6—severe.

### 2.4. Immunohistochemistry Protocol

Histopathological changes observed in vein tissue samples, conducted by three harvested techniques, were graded as follows: Grade 0—without any structural damage to the entire vein wall; disturbances, defined as grade 1, included minimal damage to the endothelial integrity with a discreet stretch of the detached intimal layer (<10%); it also included a trace defection of the perivascular fat and the connective tissue of the adventitial layer, without structural injury to the medial layer and vasa vasorum. Grade 2 included focal endothelial stretching or detaching (<25%), without damaging the intern elastic membrane and medial layer, but with mild damage to the adventitial connective tissue and focally detached vasa vasorum; this included moderate damage to the perivascular fat tissue. Grade 3 was based on moderate endothelial stretching or stripping (25–50%), in some cases focally detached, with minimal damage to the intern elastic membrane and medial layer, but with moderate damage to the adventitial tissue and vasa vasorum. Grade 4 was marked for tissue samples that showed a partially detached endothelium (>50%), damaged intimal elastic membrane, some focal areas of separated smooth muscle fibers of the medial layer, and a damaged adventitial layer with a partially absent vasa vasorum; this was also without the presence of the perivascular fat. Grade 5 included very marked changes to the luminal integrity, with a mostly stripped endothelium (>75%) and a segmentally separated intimal layer from the deeper layers; it also included damage to the intimal elastic membrane with the discontinued elastic and smooth muscle fibers of the medial layer, and extensive damage to the adventitial layer and vasa vasorum without the surrounding perivascular adipose tissue. Grade 6 was defined as vein tissue samples that had shown extensive structural damage to all three layers; this included a detached endothelial and medial layer, a stripped adventitial layer, and the absence of the vasa vasorum.

Immunohistochemical analysis was performed on routinely prepared 4 µm thick vein sections. The wall integrity of the saphenous veins was estimated using the antibodies against CD31, Factor VIII, and Caveolin, while the endothelial function was evaluated using endothelial nitric oxide synthase (eNOS). All tissue slides were examined and scored as follows: Grade 0—no staining, Grade 1—trace, Grade 2—minimal, Grade 3—moderate, Grade 4—strong.

The entire protocol of pathohistological examination is presented in Methods IV in the Supplementary Materials.

The main aim of the study was to analyzing the influence of three different surgical techniques on vein structural damage.

### 2.5. Statistical Protocol

The minimum sample size required to detect the effect size of 0.5 in the analysis of variance for the three group variables, for a level of statistical significance of 0.05 and statistical power of 0.9, is 54 respondents. The magnitude of the effect was obtained based on the assumed value ratios explained in residual variances from 0.2 to 0.98. The calculation of the sample size was performed using the program G-power 3.1.6.

The data was processed using the statistical software SPSS 25.0 for Windows 10. All statistical methods were considered significant if the *p*-value was ≤0.05. The descriptive statistics included the mean values, median, standard deviations, and the interquartile range of the parameters monitored. The difference in the distribution of specific parameters among the groups tested was determined using the χ2 or Fisher’s test. The normality of the distribution of the numerical variables was tested using the Shapiro Wilk and Kolmogorov Smirnov tests. The comparison between the groups was made using the ANOVA and the Kruskal–Wallis H test. Binominal logistic regression analysis was used to explain the relationship between the dependent binary variable and the independent variables.

## 3. Results

The study included 83 patients randomly assigned into one of the three different groups. The CVH technique was performed on 27 patients (48% male), with a mean age of 67.66 ± 5.6. The NT group included 31 patients (74% male), with a mean age of 66.5 ± 7.4. The EVH technique was performed on 25 patients (75% male). The mean age was 66.0 ± 5.5. All groups were homogenous in the preoperative characteristics.

All CABG procedures were performed using a cardio-pulmonary bypass. The internal thoracic artery was used in all participants, and additional grafts were SVG. The operation time was significantly longer in the EVH group (*p* < 0.001). One patient (3.2%) died from the NT group due to respiratory insufficiency during the early postoperative period. The incidence of the postoperative leg wound complication was significantly lower in the EVH group than in the NT and CVH groups (*p* < 0.05).

### Pathophysiological Examination

**Figure 1:**
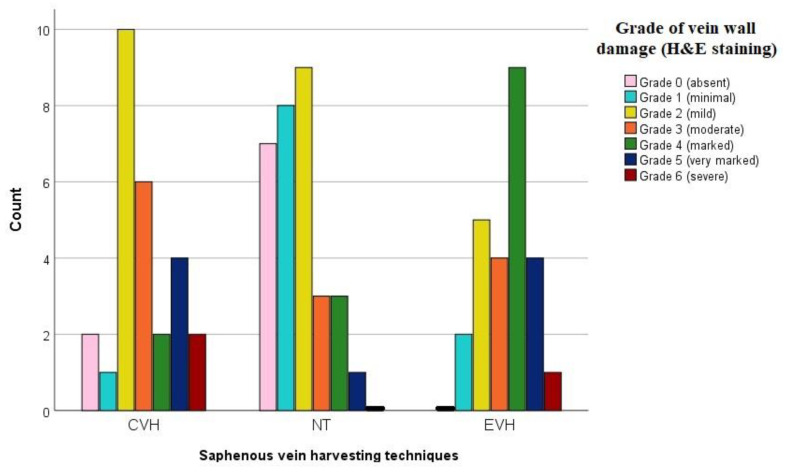
Bar chart representing grade of the vein wall damage by H&E staining in the three different groups. Abbreviations: EVH—endoscopic vein harvesting, CVH—conventional vein harvesting, NT—no-touch harvesting, H&E—hematoxylin eosin staining.

**Figure 2:**
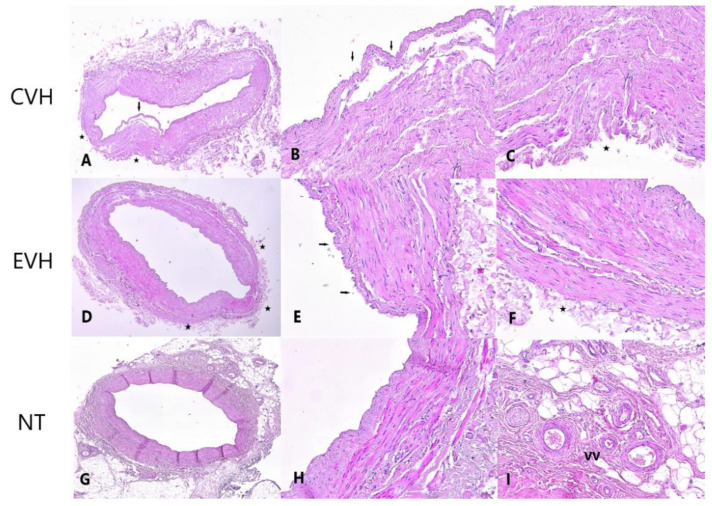
Histological presentation of the vein samples obtained by three different techniques shows detached endothelium (arrow) and focal defects of the adventitial layer with focally absent vasa vasorum (star) in CVH vein samples ((**A**)—H&E ×40; (**B**)—H&E ×200, (**C**)—H&E ×200), focal defects of the endothelial (arrows) and adventitial layer with focally absent vasa vasorum (stars) in EVH samples ((**D**)—H&E ×40; (**E**)—H&E ×200, (**F**)—H&E ×200) and intact vein wall with the vasa vasorum (VV) presence in the adventitial layer of NT vein samples ((**G**)—H&E ×40, (**H**)—H&E ×200, (**I**)—H&E ×200).

H&E staining showed a significantly lower grade of vein wall damage in the NT group (2, IQR 1-2) in comparison with CVH and EVH groups (3, IQR 2-4, and 4, IQR 2-4, respectively, *p* < 0.001). Preservation of the adventitial layer and vasa vasorum was significantly better in the NT group, compared to the CHV and EVH groups (*p* < 0.001 and *p* = 0.009, respectively), Figure 1 and Figure 2. Other characteristics of the H&E staining are presented in Supplement Table S1.

**Figure 3:**
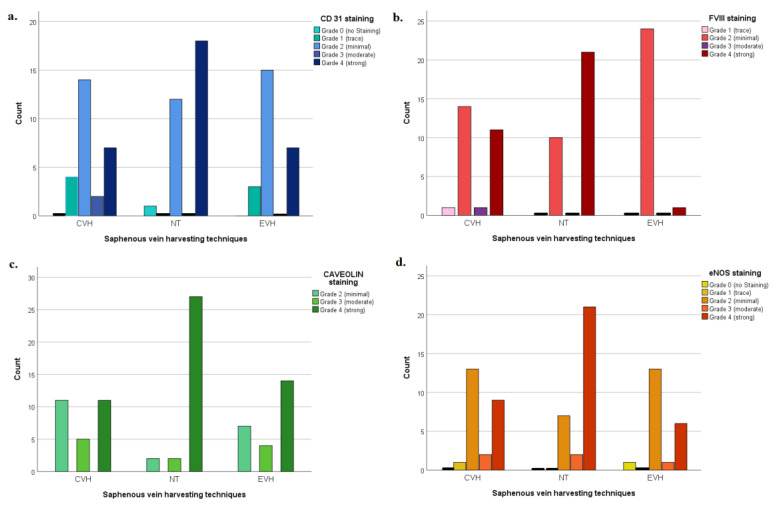
The bar chart presents patients’ distribution according to the different grades of the veins wall damage divided by groups and immunohistochemical antibodies. (**a**) CD 31 antibody staining; (**b**) Factor VIII staining; (**c**) Caveolin antibody staining; (**d**) eNOS antibody staining; Abbreviations: EVH—endoscopic vein harvesting, CVH—conventional vein harvesting, NT—no-touch harvesting.

**Figure 4:**
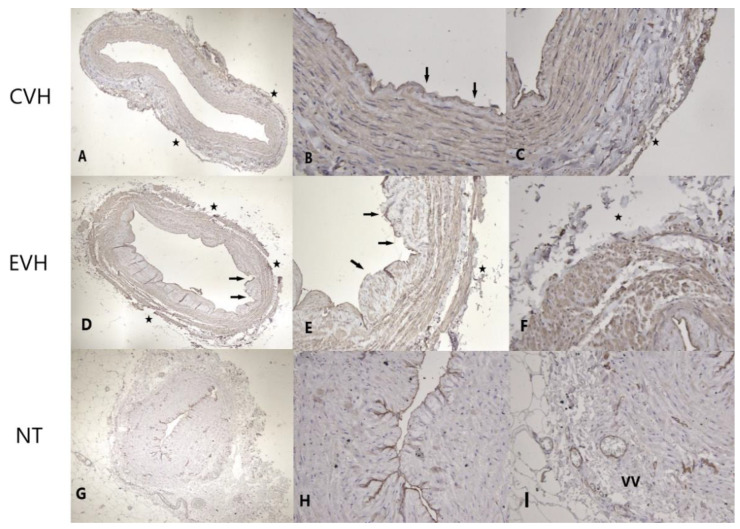
Immunohistochemical staining of the CD31 shows focally moderate staining of the endothelium (arrow) and absence of the vasa vasorum in adventitial layer (star) in CVH vein samples ((**A**)-IHH ×40; (**B**)-IHH ×200, (**C**)—IHH ×200), focally minimal staining of the endothelium (arrows), defects of the adventitial layer and absence of the vasa vasorum (stars) in EVH samples ((**D**)-IHH ×40; (**E**)-IHH ×100, (**F**)-IHH ×200) and strong staining (brown colored) of the endothelium and vasa vasorum (VV) in the adventitial layer of NT vein samples ((**G**)-IHH ×40, (**H**)-IHH ×200, (**I**)-IHH ×200).

The antibody CD31 showed a significantly higher staining grade in the NT group compared to the CVH and EVH groups (*p* = 0.02). The endothelial layer, adventitia, and vasa vasorum were better preserved in the NT group (*p* = 0.03, *p* = 0.06, *p* = 0.09, respectively), Figure 3a and Figure 4. There was no difference in the damage grade between the CVH and EVH techniques (Supplement Table S2).

**Figure 5:**
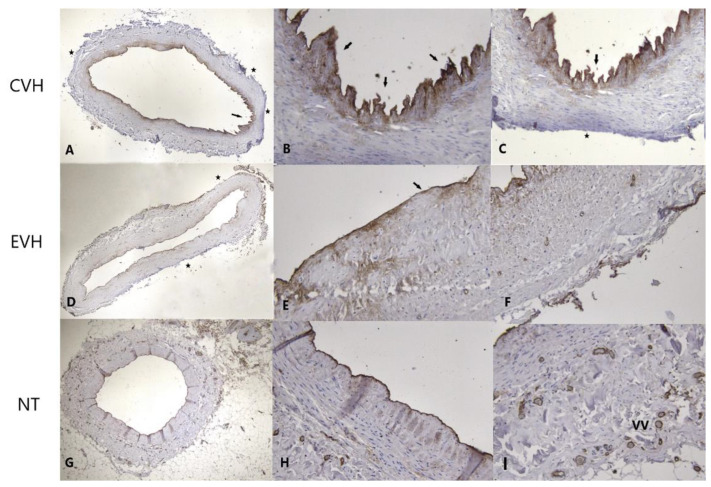
Factor VIII immunohistochemical staining illustrate strong staining (brown colored) of the focally stretched endothelium (arrow) and absence of the vasa vasorum in adventitial layer (star) in CVH vein samples ((**A**)-IHH ×40; (**B**)-IHH ×200, (**C**)-IHH ×100), focally moderate staining of the endothelium (arrows), defects of the adventitial layer and absence of the vasa vasorum (stars) in EVH samples ((**D**)-IHH ×40; (**E**)-IHH ×200, (**F**)-IHH ×200), and strong staining (brown colored) of the endothelium and vasa vasorum (VV) in the adventitial layer of NT vein samples ((**G**)-IHH ×40, (**H**)-IHH ×200, (**I**)-IHH ×200).

The factor VIII staining revealed a significantly higher grade of staining in the NT group (4, IQR 2-4) in comparison with the CVH and EVH groups (2, IQR 2-4, and 2, IQR 2-2, respectively, *p* < 0.001). The endothelial layer, adventitia, and vasa vasorum were more protected in the NT group, Figure 3b and Figure 5. Other microstructural characteristics are presented in Supplement Table S3.

**Figure 6:**
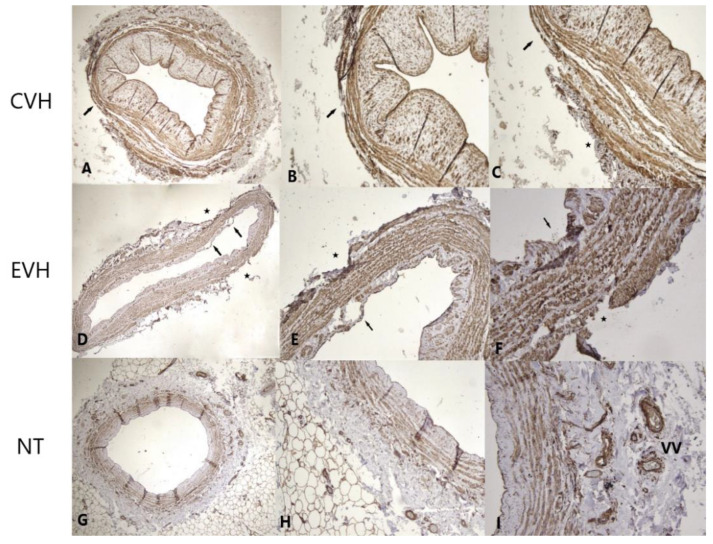
Caveolin staining of the vein samples obtained by conventional, endoscopic and no touch harvesting technique showing strong staining (brown colored) of the intimal and medial layer, illustrating focal defects of the medial (arrow) and adventitial layer (star) in CVH vein samples ((**A**)-IHH ×40; (**B**)-IHH ×100, (**C**)-IHH ×200), focal defects of the endothelial (arrow) and adventitial layer (star) in EVH samples ((**D**)-IHH ×40; (**E**)-IHH ×100, (**F**)-IHH ×200) and intact vein wall with the vasa vasorum (VV) presence in the adventitial layer of NT vein samples ((**G**)-IHH ×40, (**H**)-IHH ×100, (**I**)-IHH ×200).

The Caveolin staining showed a higher grade of staining in the NT group (4, IQR 4-4) and EVH group (4, IQR 2-4) in comparison with the CVH group (3, IQR 2-4) (*p* = 0.001). The endothelial layer, adventitia, and vasa vasorum were more protected in the NT and EVH groups than in the CVH group, Figure 3c and Figure 6. Other microstructural characteristics are presented in Supplement Table S4.

**Figure 7:**
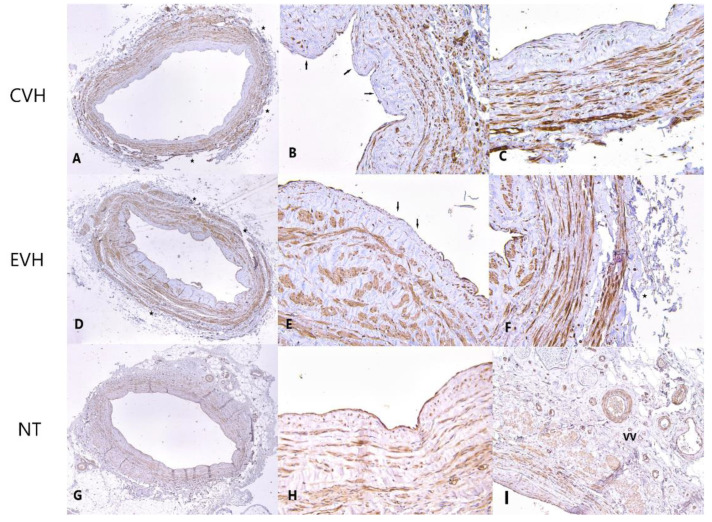
eNOS immunohistochemical staining showing focally minimal staining of the endothelium (arrow) and absence of the vasa vasorum in adventitial layer (star) in CVH vein samples ((**A**)-IHH ×40; (**B**)-IHH ×200, (**C**)-IHH ×200), minimal staining of the endothelium (arrows), focal defects of the adventitial layer and absence of the vasa vasorum (stars) in EVH samples ((**D**)-IHH ×40; (**E**)-IHH ×200, (**F**)-IHH ×200) and strong staining (brown colored) of the endothelium and vasa vasorum (VV) in adventitial layer of NT vein samples ((**G**)-IHH ×40, (**H**)-IHH ×200, (**I**)-IHH ×200).

The eNOS antibody showed a significantly higher grade of staining in the NT group (4, IQR 2.7-4) compared with CVH and EVH groups (2, IQR 2-4) and (2, IQR 2-2), respectively, (*p* = 0.003). The endothelial layer, adventitia, and vasa vasorum are better preserved in the NT group than in the others (*p* = 0.015, *p* = 0.07, and *p* = 0.02, respectively), Figure 3d and Figure 7. Other microstructural characteristics are presented in Supplement Table S10.

## 4. Discussion

The use of arterial conduits for myocardial revascularization increased during the last decade; despite this, the SVG is still the most commonly used graft due to its availability, less demanding harvesting techniques and greater resistance on the spasm than arterial grafts [8]. The most common disadvantage of the SVGs is a high graft occlusion rate. The early acute thrombosis, intimal hyperplasia, and atherosclerosis are initiated and accelerated by different factors: conduit diameter, surgical conduit preparation, conduit handling, grafting site, coronary vascular bed (run-off), patients’ risk factors, surgical skills and techniques, and technical errors [5,6,9]. The SVG harvesting strategies have evolved in two different directions: the leg complication reduction after SVG harvesting and the decrease in the graft manipulation to preserve the integrity of the SVG wall structure [10,11]. Although the surgical techniques have improved over time, the optimal strategy is still unclear [12]. We analyzed the microscopic characteristics of the SVGs harvested by three surgical techniques. H&E staining illustrates the architecture and differences between NT, CVH and EVH groups. In our study, grade of adventitial and vasa vasorum damage was significantly higher in the CVH and EVH, compared to the NT group. These are similar results to previously published studies [13,14,15]. Evidence suggests that adventitial damage is a crucial step of the adverse remodeling of the SVG after CABG [16]. Perivascular tissue and intact adventitia present a “natural external stent” that supports the vein and mitigates the negative effect of pulsatile stress, consequently decreasing the intimal hyperplasia and atherosclerosis, and potentially improving patency rate. The pedicle prevents vein kinking after implantation, decreasing the early graft failure. In addition, perivascular adipose tissue is a source of adipocyte-derived, anticontractile factors, such as nitric oxide and leptin, which may play a role in reducing spasms and maintaining graft patency [17,18]. NT graft harvesting preserved blood flow through the vasa vasorum after implantation, providing a better patency rate over the CVH [19,20]. H&E staining did not show differences in the endothelial damage between the groups compared in our study.

On the other hand, immunohistochemical staining by CD31, vWF, Caveolin, and eNOS showed a significantly higher grade of endothelial damage in the CVH and EVH groups compared to the NT group. These results are similar to the other studies, which concluded that excessive manipulation, blunt trauma of the SVGs, graft flushing and distension with uncontrolled pressure, impaired the integrity and function of the endothelium [7,21,22]. Dissecting and striping a vein using a CVH and EVH can result in vascular spasm and graft wall damage, leading to graft occlusion. The NT technique prevents vein spasms and excludes manual dilatation of the SVG, consequently increasing graft preservation, compared with other harvesting techniques [23]. Hwang et al. showed a lower grade of endothelial destruction in the harvesting group with minimal vein manipulation, compared to the conventional technique [24]. Saito T. et al. claim that vein flashing, with a pressure less than 100 mmHg, reduced CD31 staining by half, while increasing the pressure produced a higher level of endothelial damage [25]. In a multicenter randomized controlled trial on a no-touch technique for vein harvesting in CABG, Tian et al. reported >40% relative reductions in the rate of saphenous vein graft occlusions, compared to the conventional method. The main disadvantage of the NT harvesting is the higher rate of LW dehiscence, due to a significant subcutaneous defect and the consequent increased tension on the suture line. Therefore, the wound reconstruction must be meticulous, and results could be improved through dedicated training [26]. The cohort study included 3000 patients who underwent the CABG procedure with SVGs, and revealed that EVH is independently associated with vein-graft failure and adverse clinical outcomes [27]. The randomized study, published in the New England of Medicine 2019, did not reveal a significant difference between open vein-graft harvesting and endoscopic vein-graft harvesting in relation to the risk of major adverse cardiac events [28]. The appropriate selection of patients, surgical skills, and education, according to proctor instructions, are keystones for good-quality, long lasting SVGs.

### 4.1. Study Limitation

A higher number of patients is required to increase the study power. The correlation between the degree of graft damage and the early patency rate controlled by computer tomography was not analyzed.

### 4.2. Futured Research

The relationship between the degree of VSGs wall damage, and early and long-term graft failure rate in the mentioned harvesting techniques, could be an interesting future study.

## 5. Conclusions

Immunohistochemical analyses showed that the best microstructural preservation of the SVGs is achieved by NT harvesting, while the EVH technique significantly reduced the leg wound complication. The development and implementation of the endoscopic no-touch technique could be the best strategy option.

## Data Availability

All the data are available from the corresponding author upon reasonable request.

## Supplementary Materials

The following supporting information can be downloaded at: https://www.mdpi.com/article/10.3390/medicina59020217/s1, Figure S1: CD31 staining of the endothelial layer; A—preserved endothelial cells; B—destructed endothelial cells; Figure S2: Caveolin staining of the tunica media; A—preserved smooth muscle; B—detachment of the smooth muscle. Figure S3: Caveolin staining od the tunica adventitia; A—preserved adventitia and vasa vasorum; B—destructed adventitia and vasa vaso-rum; Table S1: The vein wall damage detected by immunohistochemical Haematoxylin eosin staining.; Table S2: The vein wall damage detected by immunohistochemical CD31 staining; Table S3: The vein wall damage detected by immunohistochemical Factor VIII staining; Table S4: The vein wall damage detected by immunohistochemical Caveolin staining; Table S5: The vein wall damage detected by immunohistochemical eNOS staining.

## Abbreviations

CABG—coronary artery bypass grafting
CAD—coronary artery disease
CVH—conventional vein harvesting
eNOS—endothelial nitric oxide synthase
EVH—endoscopic vein harvesting
H&E—haematoxylin eosin
IQR—interquartile range
LITA—left internal thoracic artery
LAD—left anterior descending artery
LW—leg wound
MI—myocardial infarction
NT—no-touch harvesting
PCI—percutaneous coronary intervention
SVG—saphenous vein graft
