# Supplementary material for: Influence of Three Different Surgical Techniques on Microscopic Damage of Saphenous Vein Grafts—A Randomized Study
Source: Medicina (Kaunas). 2023 Jan 23;59(2):217. doi: 10.3390/medicina59020217 (PMC9962261; doi:10.3390/medicina59020217)
Supplement: Supplementary file 1 [file medicina-59-00217-s001.zip › medicina-2057478-supplementary.pdf]

SUPPLEMENTARY MATERIAL

Content:

I – inclusion and exclusion criteria
II – preoperative ultrasound great saphenous vein mapping
III – surgical procedure and vein grafts harvesting
IV – pathohistological and immunohistochemical protocols
V – tables

I. Inclusion and exclusion criteria

The candidates for this study were all patients who planned to undergo primary open-chest CABG with at least one great saphenous vein graft. Patients eligible for the study had to fulfil the following criteria:

Vein grafts harvested by one highly experienced cardiac surgeon (more than 100 vein grafts per year)

CABG with at least one great saphenous vein graft.

According to preoperative ultrasound vein mapping, the acceptable vein diameter was 2–5 mm.

Patients who fulfilled any of the following criteria were ineligible for study:

Combined cardiac surgical procedure

Redo CABG

Emergent patients due to STEMI or NSTEMI

Failing of percutaneous coronary intervention and hemodynamic disturbance

Severe peripheral vascular disease

Patients planned for total arterial revascularization

Coronary revascularization used multiple arterial grafts

Previous vein stripping

Dilated or varicose great saphenous vein revealed by ultrasound vein mapping

I. Preoperative ultrasound great saphenous vein mapping

According to our clinical protocol, vein mapping is performed one day before the CABG procedure. The patient is on the bed in the supine position, both legs a complete exposure for examination. We used a general electric ultrasound device and a 9L – D linear probe. The entire length of the great saphenous vein of both legs was assessed and marked using a water-proof skin marking pen during the mapping process. The vein diameter was assessed and measured at multiple sites. The acceptable diameter in our study was between 2 and 5 mm. Large tributaries, varicosities and duplicate systems are detected and marked with a skin landmark. The duration of the mapping was not more than 10 min per case. The preoperative ultrasound vein mapping aimed to detect the best segment we will use as a graft for revascularization.

I. Surgical procedure and vein graft harvesting

According to the institutional protocols, all patients underwent CABG under general anesthesia. Cardiopulmonary bypass and systemic heparinization were used in all participants. An antegrade cold blood cardioplegic solution performed myocardial protection.

CABG procedures were performed by highly experienced cardiac surgeons with more than ten years of experience, who performed at least 50 CABG procedures per year. Vein harvesting was performed by one high qualified cardiac surgeon with six years of experience. The surgeon had performed more than 100 vein grafts using conventional techniques per year. According to the proctor's instructions, the same surgeon had harvested more than 50 no-touch vein grafts (the proctor was Dr. Domingos Souza, who developed this technique). The surgeon had extensive experience in endoscopic vein harvesting and performed more than 50 cases (He is a Terumo's proctor for endoscopic vein and radial harvesting).

According to preoperative mapping, the best segment of the great saphenous vein was harvested.

For the conventional technique, longitudinal skin and subcutaneous tissue incisions were made. After exposure and hemostasis, a vein was sharply dissected from the perivascular tissue. The side branches were ligated using a 4-0 silk or clipped by a metal clip. When we harvested enough length, a vein was cut, and in the open distal end of the vein, a small metal cannula was inserted and secured with ligation. The vein was distended by a syringe with a manometer (pressure about 200mmHg), using the heparinized saline solution and stored in the same solution.

In the no-touch group, a complete longitudinal skin incision was made. The subcutaneous tissue dissection was performed using low power diathermy. The most important task is to keep the fascia and perivascular tissue intact. A vein was harvested with a 1 cm wide perivascular fat pedicle. The side branches were carefully dissected and ligated by 4-0 silk or metal clips. After reaching the needed length, the vein was removed, and in the open distal, a small metal cannula was inserted and secured by ligation. Forced distension or flushing using a syringe was strictly prohibited. The vein cannula was connected to the line from the cannula inserted into the ascending aorta. The systemic tension heparinized blood was used for vein distension, flashing and for checking the bleeding. The grafts were preserved in the heparinized saline solution for the implantation.

The endoscopic vein harvesting was performed using the VirtuSaph Plus open CO2 system and endoscope device, devised by Richard Wolf.

In order to enable the correct control of the device, the selected leg is positioned so that a roughly 90° angle is formed between the thigh and leg. According to the mapping landmark, the 1–2 cm longitudinal incision was made just below the knee, at the inner margin of the gastrocnemius muscle. The vein was dissected via direct visualization and gently hooked with silicone tape. The next step in the harvesting process was performed by a PTFE coated dissector with an optical probe and CO2 line (CO2 flow rate: 3L/min, and maximum pressure in the insufflator: 12 mmHg). The dissector creates a tunnel around the vein, the first on the posterior side of the vein and then on the upper face. The side branches are prepared by gently dissecting them. After the complete dissection of the vein and side branch, the dissector is pulled out, and the endoscope and CO2 are inserted and connected to the harvester. All side branches were cut by V cut bipolar diathermy. When all side branches had been cut, the small skin incision was made near the vein graft's proximal end. The blood vessel was gripped, pulled out through the skin hole, tied, and cut. The harvesting process was performed without systemic heparinization. A small metal cannula was inserted into the distal end of the vein and secured with ligation. The vein was distended by a syringe with a manometer (pressure about 200 mmHg) using the heparinized saline solution, and stored in the same solution. All branches are ligated with 4-0 silk ties; small avulsions may be repaired using 7-0 polypropylene purse-string sutures.

The subcutaneous incisions in the first-mentioned techniques were closed by continuous Vicryl (undyed braided) suture in one or two layers, depending on the subcutaneous tissue thickness. The skin incisions were close, according to the surgeon's preferences (intradermal or interrupted suture). In the endoscopic group, only the skin suture was used.

I. Pathohistological protocol

The vein samples were numerically coded to ensure laboratory blinding. The 1 cm long saphenous vein samples were fixed in 4% buffer formalin solution (Immersion fixation) and molded into paraffin, processed routinely. The paraffin blocks were sectioned at a thickness of 4 μm, then deparaffinized, rehydrated, and stained with hematoxylin and eosin (H&E), following by standard protocols. Stained vein samples taken from each patient were observed using a light microscope Olympus BX50 (Olympus, Japan), equipped with a digital camera Leica DFC 295 (Leica Microsystems,

Germany) under different magnifications. For each patient, five slides were analyzed. All vein samples were observed for the structure changes with a detailed examination of the entire H&E section for each patient by two independent pathologists. The extent of vein wall changes was based on the estimation of all three layers: tunica intima, media, and adventitia. Histological evaluation was directed to estimate the endothelial integrity and the degree of the degenerative changes in the elastic fibers, smooth muscle, and connective tissue. The observed pathohistological changes were graded as follows: ***absent*** (0), ***mild*** (1), ***mild to moderate*** (2), ***moderate*** (3), ***moderate to severe*** (4) and ***severe/significant*** (5).

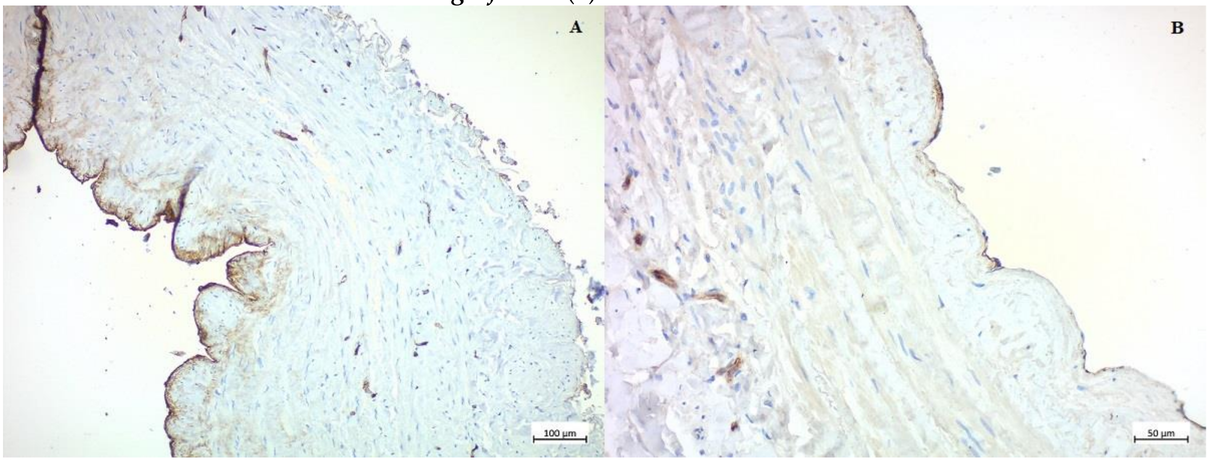

Figure S1. CD31 staining of the endothelial layer; A – preserved endothelial cells; B – destructed endothelial cells.

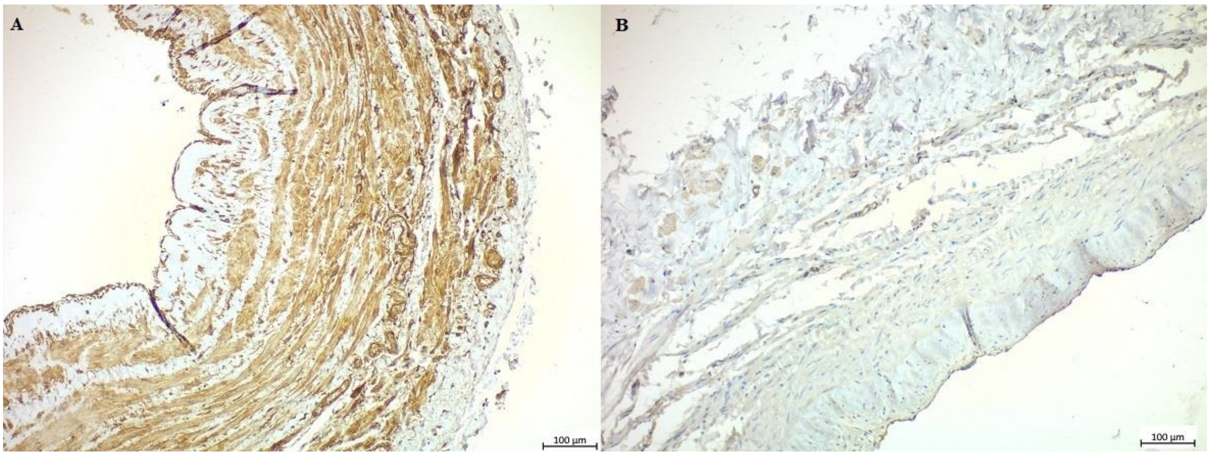

Figure S2. Caveolin staining of the tunica media; A – preserved smooth muscle; B – detachment of the smooth muscle.

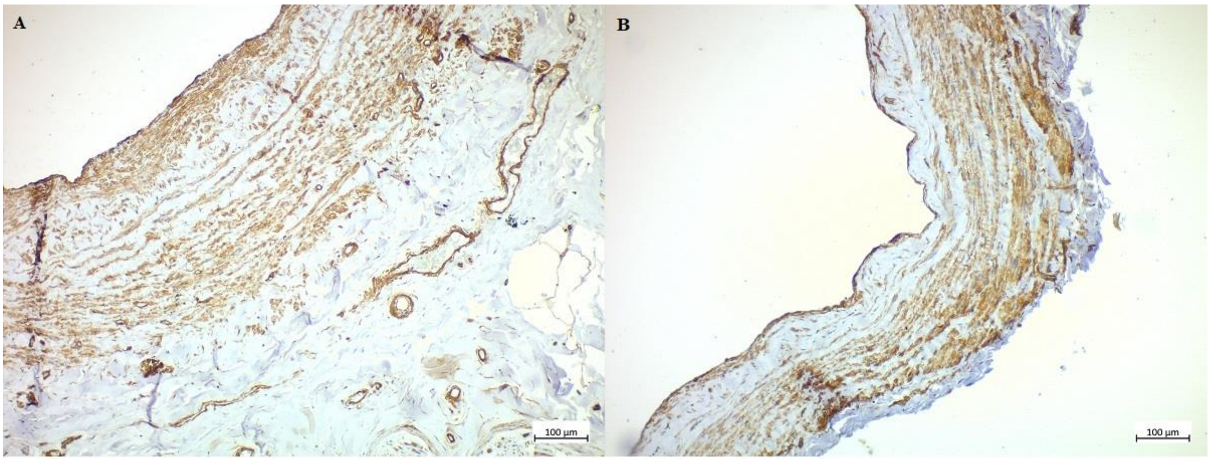

Figure S3. Caveolin staining od the tunica adventitia; A – preserved adventitia and vasa vasorum; B – destructed adventitia and vasa vasorum.

Immunohistochemistry protocol

An immunohistochemical study was performed using the Ultravision LP – HRP polymer detection technique. For the immunohistochemical evaluation of wall integrity, mouse monoclonal antibodies were used against human endothelial glycoprotein CD31 (Dako FLEX IS610), rabbit polyclonal von Willebrand factor (Dako FLEX IS527), and rabbit polyclonal antibody against caveolin (diluted 1:350, Abcam ab2910). In contrast, rabbit polyclonal antibodies, were used against endothelial nitric oxide synthase – eNOS (diluted 1:200, Abcam ab5589) to examine the endothelial function.

Procedure

Immunohistochemical staining was performed on formalin-fixed, paraffin-embedded 3μm thick vein sections. After the deparaffinization and inhibition of the endogenous peroxidase activity, the procedure was continued by uncovering the antigen, exposing the tissue sections to a high temperature and immersing them in a suitable buffer (Table). Then the tissue sections were incubated with the primary antibody into a humid chamber and then rinsed in TBS (Tris buffer solution) three times for three minutes to block background staining. After washing, sections were incubated with **the Primary Antibody Enhancer** kit component. The next step was incubation with HRP Polymer reagent. The procedure continued with the incubation of the tissue sections in a substrate–chromogen mixture solution (3,3-diaminobenzidine–DAB, Dako Liquid DAB+ Substrate Chromogen System, K3467), rinsed with tap water and counterstaining with Mayer's hematoxylin. After that, tissue sections were dehydrated, and covered with synthetic resin and cover glass. With this final step, slides were prepared for detailed microscopic examination.

Note

As **HRP Polymer** is photosensitive, it was necessary to avoid exposure to the light.

Interpretation of the results: If the examined antigen is present, a precipitate of chromogen is observed at the site as a brown color, in contrast to the surrounding tissue colored blue.

Quality control and specifics of immunohistochemical reaction

During the staining process, both "positive" and "negative" control samples were stained to confirm the specificity and quality of the immunohistochemical method. As a positive control, tissue samples that have previously been reliably determined to contain antigens can be visualized by the applied method. These sections are treated in the same way as the examined tissue samples. On the other hand, the negative control (reagent control) was represented by tissue samples with non-immune serum, instead of primary antibodies.

Table. Details about monoclonal and polyclonal antibodies and its preparation for immunohistochemical staining

Primary antibody	Manufacturer / Catalogue number	Dilution	Preincubation	Incubation	Kit	Manufacturer / Catalogue number
Rb* Anti-Caveolin 1 antibody -	Abcam, ab2910	1:350	Epitope retrieval solution pH9, 20 minutes in microwave	30 minutes on room temperature	Ultravision LP- HRP polymer	Thermoscientific / Cat. No. TL-125-HL

Caveolae Marker, polyclonal						
Rb polyclonal Anti-eNOS antibody	Abcam, ab5589	1:200	0,01M citrated buffer pH6, 20 minutes in microwave	overnight at +4˚C	Ultravision LP- HRP polymer	Thermoscientific / Cat. No. TL-125-HL
Rb polyclonal Factor VIII	DAKO FLEX IS527	RTU***	Epitope retrieval solution pH9, 20 minutes in microwave	30 minutes on room temperature	Ultravision LP- HRP polymer	Thermoscientific / Cat. No. TL-125-HL
Mo** monoclonal Anti-CD31, Endothelial Cell, Clone JC70A	DAKO FLEX IS610	RTU	Epitope retrieval solution pH9, 20 minutes in microwave	30 minutes on room temperature	Ultravision LP- HRP polymer	Thermoscientific / Cat. No. TL-125-HL

*Rb – Rabbit; **Mo – Mouse; ***RTU – Ready-to-Use.

Table S1. The vein wall damage detected by immunohistochemical Haematoxylin eosin staining.

Hematoxylin Eosin staining	CVH group N(27)	NT group N(31)	EVH group N(25)	P value
Grade of vein wall damage	3 (IQR 2-4)	2 (IQR 1-2)	4 (IQR 2-4)	**<0.001**
Endothelial damage	7 (25.9%)	8 (25.8%)	7 (28%)	0.98
Medial layer damage	5 (18.5%)	7 (22.5%)	4 (16%)	0.8
Intimal hyperplasia	5 (18.5%)	10 (32.25%)	10 (40%)	0.23
Adventitia damage (0 -no, 1- local, 2 – diffuse)	7 (25.9%)<br>14 (51.8%)<br>6 (22.2%)	25 (80.6%)<br>6 (19.3%)<br>0 (0%)	5 (20%)<br>15 (60%)<br>5 (20%)	**<0.001**
Vasa vasorum damage	6 (22.2%)	0 (0%)	7 (28%)	**0.009**

Abbreviations: CVH – conventional vein harvesting, EVH – endoscopic vein harvesting, IQR – interquartile range, NT – no touch harvesting.

Table S2. The vein wall damage detected by immunohistochemical CD31 staining.

CD31 staining	CVH group N(27)	NT group N(31)	EVH group N(25)	P value
Grade of staining	2 (IQR 2-4)	4 (IQR 2 -4)	2 (IQR 2-4)	**0.02**
Endothelial damage (0 – no, 1-lokal, 2 – diffuse)	6 (22.2%)<br>17 (62.96%)<br>4 (14.8%)	16 (51.6%)<br>8 (25.8%)<br>6 (19.3%)	7 (28%)<br>16 (64%)<br>2 (8%)	**0.03**
Medial layer damage	2 (7.4%)	1 (3.22%)	1 (4%)	0.75
Adventitia damage (0 – no, 1- local, 2 – diffuse)	19 (70.3%)<br>7 (25.9%)<br>1 (3.7%)	30 (96.77%)<br>1 (3.22%)<br>0 (0%)	22 (88%)<br>2 (8%)<br>1 (4%)	**0.06**
Vasa vasorum damage	4 (14.8%)	0 (0%)	3 (12%)	**0.09**

Abbreviations: CVH – conventional vein harvesting, EVH – endoscopic vein harvesting, IQR – interquartile range, NT – no touch harvesting.

Table S3. The vein wall damage detected by immunohistochemical Factor VIII staining.

Factor VIII	CVH group N(27)	NT group N(31)	EVH group N(25)	P value
Grade of staining	2 (IQR 2-4)	4 (IQR 2-4)	2 (IQR 2-2)	**<0.001**
Endothelial damage (0-no, 1-local, 2 – diffuse)	27 (100%)<br>0<br>0	30 (96.7%)<br>1 (3.22%)	21 (84%)<br>4 (16%)	**0.038**
Medial layer damage	0 (0%)	0 (0%)	0 (0%)	0.99
Adventitia damage (0 -no, 1- local, 2 - diffuse)	26 (96.29%)<br>1 (3.7%)	31 (100%)	25 (100%)	0.35
Vasa vasorum damage	1 (3.7%)	0 (0%)	0 (0%)	0.35

Abbreviations: CVH – conventional vein harvesting, EVH – endoscopic vein harvesting, NT – no touch vein harvesting.

Table S4. The vein wall damage detected by immunohistochemical Caveolin staining.

Caveolin staining	CVH group N(27)	NT group N(31)	EVH group N(25)	P value
Grade of staining	3 (IQR 2-4)	4 (IQR 4-4)	4 (IQR 2-4)	**0.001**
Endothelial damage (0 - no, 1-local, 2 – diffuse)	14 (51.8%)<br>10 (37%)<br>3 (11.1%)	22 (70.9%)<br>8 (25.8%)<br>1 (3.22%)	12 (48%)<br>13 (52%)<br>0 (0%)	**0.01**
Medial layer damage	7 (25.9%)	2 (6.4%)	0 (0%)	**0.007**
Adventitia damage (0 - no, 1- local, 2 - diffuse)	24 (88.8%)<br>2 (7.4%)<br>1 (3.7 %)	31 (100%)<br>0 (0%)<br>0 (0%)	23 (92%)<br>0 (0%)<br>2 (8%)	0.15
Vasa vasorum damage	4 (14.8%)	0 (0%)	3 (12%)	**0.09**

Abbreviations: CVH – conventional vein harvesting, EVH – endoscopic vein harvesting, IQR – interquartile range, NT – no touch harvesting.

Table S5. The vein wall damage detected by immunohistochemical eNOS staining.

eNOS staining	CVH group N(27)	NT group N(31)	EVH group N(25)	P value
Grade of staining	2 (IQR 2-4)	4 (IQR 2.7-4)	2 (IQR 2-2)	**0.003**
Endothelial damage (0 - no,1-local, 2 – diffuse)	9 (33.3%)<br>9 (33.3%)<br>4 (14.8%)	15 (48.4%)<br>4 (12.9%)<br>2 (6.45%)	3 (12%)<br>9 (36%)<br>6 (24%)	**0.015**
Medial layer damage	1 (3.7%)	0 (0%)	0 (0%)	0.4
Adventitia damage (0 – no, 1- local, 2 – diffuse)	11 (40.7%)<br>8 (29.6%)<br>3 (11.1%)	19 (61.3%)<br>2 (6.45%)<br>0 (0%)	11 (44%)<br>5 (20%)<br>2 (8%)	**0.07**
Vasa vasorum damage	6 (22.2%)	0 (0%)	6 (24%)	**0.02**

Abbreviations: CVH – conventional vein harvesting, EVH – endoscopic vein harvesting, IQR – interquartile range, NT – no touch harvesting.
